# Fungal Laccases with High and Medium Redox Potential: Is the T1 Center Potential a Key Characteristic of Catalytic Efficiency in Heterogeneous and Homogeneous Reactions?

**DOI:** 10.3390/ijms26157488

**Published:** 2025-08-02

**Authors:** Olga Morozova, Maria Khlupova, Irina Vasil’eva, Alexander Yaropolov, Tatyana Fedorova

**Affiliations:** A. N. Bach Institute of Biochemistry, Research Center of Biotechnology, Russian Academy of Sciences, Leninsky Ave. 33, 119071 Moscow, Russia; dave80@yandex.ru (M.K.); ir-vas@yandex.ru (I.V.); yaropolov@inbi.ras.ru (A.Y.)

**Keywords:** biocatalysis, high and medium redox potential laccases, homogeneous catalysis, mediatorless bioelectrocatalysis

## Abstract

Catalytic and bioelectrocatalytic properties of four white rot fungal laccases (*Trametes hirsuta*, ThL; *Coriolopsis caperata*, CcL; *Steccherinum murashkinskyi*, SmL; and *Antrodiella faginea*, AfL) from different orthologous groups were comparatively studied in homogeneous reactions of electron donor substrate oxidation and in a heterogeneous reaction of dioxygen electroreduction. The ThL and CcL laccases belong to high-redox-potential enzymes (E^0^_T1_ = 780 mV), while the AfL and SmL laccases are medium-redox-potential enzymes (E^0^_T1_ = 620 and 650 mV). We evaluated the efficiency of laccases in mediatorless bioelectrocatalytic dioxygen reduction by the steady-state potential (E_ss_), onset potential (E_onset_), half-wave potential (E_1/2_), and the slope of the linear segment of the polarization curve. A good correlation was observed between the T1 center potential of the laccases and their electrocatalytic characteristics; however, no correlation with the homogeneous reactions of electron donor substrates’ oxidation was detected. The results obtained are discussed in the light of the known data on the three-dimensional structures of the laccases studied.

## 1. Introduction

The emerging environmentally friendly technologies employing biocatalysts rather than traditional chemical catalysts has attracted increasing attention. Laccases (*p*-diphenol/oxygen oxidoreductase, EC 1.10.3.2), belonging to the blue multicopper oxidase (MCO) family, are promising biocatalysts capable of biotransforming various compounds. Laccases are non-specific enzymes that catalyze the single-electron oxidation of numerous organic compounds, such as monophenols, polyphenols, and aromatic amines, as well as certain inorganic compounds, with the concomitant reduction in molecular oxygen to water [1,2]. However, despite extensive research, the molecular basis of their functional diversity still remains unclear. In particular, no current method can reliably predict laccase activity towards complex substrates [3]. This presents a challenge to the rational design of laccases for various technological applications.

Fungal laccases comprise three cupredoxin-type domains with a Greek-key β-barrel topology. The active site of laccases includes four copper ions, typically classified as T1, T2, and T3 copper centers. The T2 and T3 centers form a trinuclear copper cluster (TNC) [4]. The T1 center is located in the third domain, while the T2/T3 cluster is between the first and third domains. The T1 center is localized at a distance of ~12–13 Å from TNC and is connected to it by a conserved His-Cys-His triad involved in electron transfer [5]. Based on the T1 center redox potential, laccases are subdivided into high- (E^0^_T1_ = 720–790 mV), medium- (E^0^_T1_ = 460–720 mV), and low-potential (E^0^_T1_ ≤ 460 mV) enzymes [2,6]. The T1 center is thought to accept electrons from reducing substrates; the electrons are transferred to TNC where molecular oxygen is reduced to water [4,7].

Laccases are promising biocatalysts which can be used in various applications [8]. They can serve as electrocatalysts for the dioxygen reduction reaction [6,8]. Direct (mediatorless) bioelectrocatalysis was first demonstrated for the *Polyporus versicolor* laccase adsorbed on a carbon black electrode [9]. The adsorbed laccase catalyzed dioxygen electroreduction at slightly acidic pH values in the absence of a redox mediator. The potential on the electrode in the dioxygen atmosphere was set close to the oxygen equilibrium potential. The mechanism of this bioelectrocatalytic reaction was studied by Tarasevich et al. [10], who demonstrated that the reduction of dioxygen to water occurs without the intermediate formation of hydrogen peroxide.

Recently, numerous studies have demonstrated direct bioelectrocatalysis by laccases from various sources [11,12,13,14,15], as well as by other redox enzymes such as peroxidases [16,17], hydrogenases [18,19], PQQ dehydrogenases [20,21,22], nitrite reductase [23], bilirubin oxidase [24], and cellobiose dehydrogenases [25,26]. During direct bioelectrocatalysis, enzyme-modified electrodes can act as either electron donors or acceptors, depending on the biocatalyst used.

In the case of MCOs, namely laccases and bilirubin oxidases, the electrode acts as an electron donor in bioelectrocatalytic dioxygen reduction. Using the MCO-modified carbonaceous electrodes, electrons are transferred from the electrode first to the T1 center, and then to the T2/T3 cluster where O_2_ reduction occurs [4,7,27]. Sekretareva et al. [28] considered the possibility of electron transfer from the graphite electrode directly to the T2/T3 cluster of adsorbed bilirubin oxidase, and showed that due to the high reorganization energy of the fully oxidized TNC, electron transfer from the electrode to the TNC does occur through the T1 center.

Electrocatalytic studies of laccases adsorbed on the electrode surface greatly contribute to understanding their action mechanism and estimating their efficiency in heterogeneous reactions. In general, controlling the electrode potential enables the rate of interfacial electron transfer to be changed. The electrocatalytic properties of laccases in heterogeneous dioxygen reduction depend on the enzyme T1 center’s redox potential and structure, electrode material, the enzyme orientation on its surface, and the activity of an immobilized enzyme [29,30,31,32,33,34,35].

In this work, we compared four fungal laccases from different orthologous groups to establish the relationship between the redox potential of the enzyme’s T1 center and its catalytic efficiency in homogeneous and heterogeneous reactions. To this end, we chose the laccases from the basidial white rot fungi *Coriolopsis caperata* (CcL), *Trametes hirsuta* (ThL), *Antrotiella faginea* (AfL), and *Steccherinum murashkinskyi* (SmL).

## 2. Results and Discussion

The most studied and well-characterized laccases are produced by white rot fungi belonging to the order Polyporales. The T1 center of many laccases, mostly represented by the *Polyporaceae* family, has a high redox potential, which enables the efficient oxidation of numerous substrates and makes laccases promising biocatalysts. However, the catalytic properties of laccases may greatly vary, likely due to differences in their amino acid sequences, in particular, in positions critical for substrate binding. Indeed, the genomes of white rot Polyporales may contain up to nine non-allelic copies of the genes encoding laccase isoenzymes [36]. The phylogenetic analysis of laccase amino acid sequences has shown the presence of orthologous groups [36,37]. The enzymes from the same group have similar physicochemical, biochemical, and catalytic properties [38].

In the present work, we analyzed the laccases of the basidial white rot fungi from two clades of the Polyporales order: the Core (CcL and ThL) and Residual (SmL and AfL) polyporoid clades [39]. Their physicochemical properties are presented in Table 1. While all the enzymes contain four copper ions and give UV-visible absorption spectra typical of laccases, they have different carbohydrate contents, isoelectric points, and T1 center redox potentials. The CcL and ThL from the Core polyporoid clade are high-redox-potential enzymes (E^0^_T1_ = 780 mV), whereas the AfL and SmL from the Residual polyporoid clade are medium-redox-potential enzymes (E^0^_T1_ = 620 and 650 mV).

**Figure 1:**
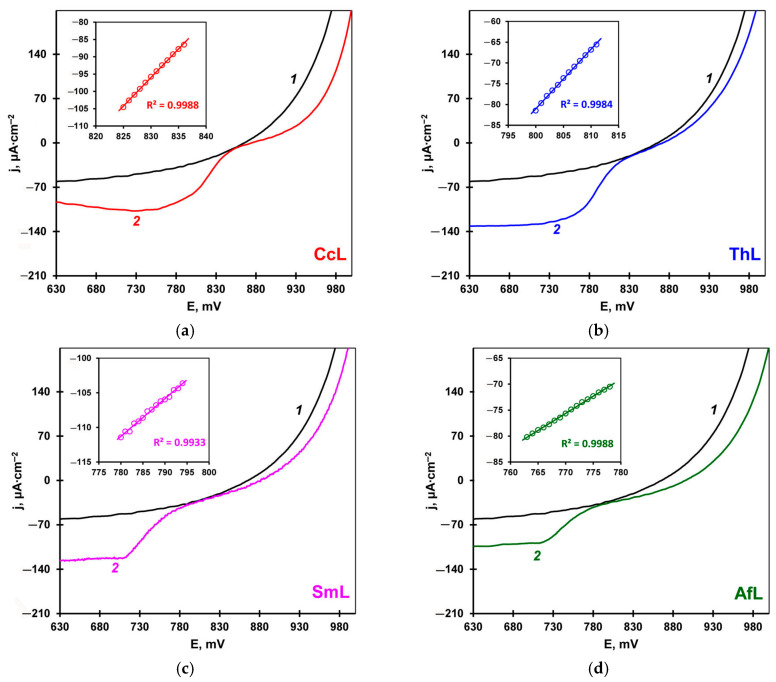
Linear voltammograms of dioxygen electroreduction on the bare (*1*) and laccase-modified (*2*) MWCNT-electrodes: (**a**) *C. caperata*, (**b**) *T. hirsuta*, (**c**) *S. murashkinskyi*, and (**d**) *A. faginea*. Insert: the linear segment of the polarization curve. Conditions: 0.1 M Na–citrate–phosphate buffer; pH 5.5; electrode working surface: 0.25 cm^2^; scan rate: 1 mV s^−1^.

All four laccases adsorbed on MWCNT-electrodes catalyzed the dioxygen electroreduction in the pH range of 3.0–5.5. Dioxygen electroreduction produced water without forming hydrogen peroxide. The analytical method using peroxidase and ABTS showed no hydrogen peroxide in the reaction medium at the potentials corresponding to the diffusion-limited current. It should be noted that all the enzymes exhibited no electrocatalytic activity at pH 7.0. Linear voltammograms (Figure 1) were recorded at a potential scan rate of 1 mV s^−1^, as at this scan rate the dioxygen reduction occurred under pseudo-steady-state conditions. In general, the polarization curve of the dioxygen electroreduction reaction includes three segments: (1) a kinetic segment characterizing the catalytic properties of an electrocatalyst; (2) a kinetic diffusion segment; (3) a diffusion-controlled segment describing the dependence of the current on the substrate mass transfer [42]. The efficiency of electrocatalysis can be estimated by the steady-state potential of an electrode (E_ss_), the onset potential (E_onset_), the half-wave potential (E_1/2_), as well as by the slope of the polarization curve linear segment close to the steady-state potential (Δi/ΔE), which is related to the exchange current density. These parameters are commonly used to assess the activity of electrocatalysts. It should be noted that the definitions of the E_onset_ vary from publication to publication [43]. We used the definition of the onset potential as a potential value corresponding to 5% of the diffusion-limited current [44].

In our work, we evaluated the efficiency of direct bioelectrocatalysis using all four parameters. Figure 1 shows linear voltammograms of dioxygen electroreduction on the MWCNT-electrodes with adsorbed enzymes recorded at pH 5.5. Table 2 summarizes the electrocatalytic characteristics of the enzymes. Still, one should bear in mind that efficient bioelectrocatalysis requires the maximum filling of the electrode surface with an enzyme in an orientation that ensures the involvement of all immobilized molecules in the bioelectrocatalytic process. The surface concentrations (Γ) of adsorbed laccases on the MWCNT-electrodes were in the range of 1.58–1.86 pmol cm^−2^ (Table 2).

As shown in Table 2, the E_onset_ and E_ss_ potentials of the electrodes modified with the high-redox-potential CcL and ThL were the closest to the equilibrium oxygen potential for O_2_/H_2_O (890 mV at pH 5.5 in the air). The medium-redox-potential SmL and AfL had more negative E_ss_, E_onset_, and E_1/2_ values, indicating a lower electrocatalytic activity in dioxygen reduction.

For most practically important electrochemical reactions, the current depends exponentially on the potential range shift:

(1) η=a+b·lgi

where η—the cathode overpotential of dioxygen electroreduction; $a=-\frac{RT}{\alpha nF}ln\left(i_{0}\right)$; $b=\frac{2.3RT}{\alpha nF}$; i—the current; i_0_—the exchange current; R—the universal gas constant; F—the Faraday constant; T—the absolute temperature; n—the number of electrons transferred; and α—the charge transfer coefficient, varying from 0 to 1. However, the initial (kinetic) segment of the polarization curve displays a linear dependence between the overvoltage and the current:

(2) $$\eta =\frac{RTi}{nFi_{0}}$$

The slope of the linear segment of the polarization curve (Δi/ΔE) correlates with the exchange current, which also characterizes the electrocatalyst activity. The steeper the slope is, the greater is the exchange current, and, consequently, the higher the activity of an electrocatalyst. As can be seen from Table 2, the Δi/ΔE values for the high-redox-potential CcL and ThL are greater compared to the medium-redox-potential SmL and AfL. The correlation between the laccase T1 center potential and the electrocatalytic characteristics (E_ss_, E_onset_, E_1/2_, and Δi/ΔE) was linear with R^2^ = 0.84–0.92 (Supplementary Figure S1). This indicates the contribution of the T1 center redox potential to the efficiency of mediatorless bioelectrocatalysis.

**Figure 2:**
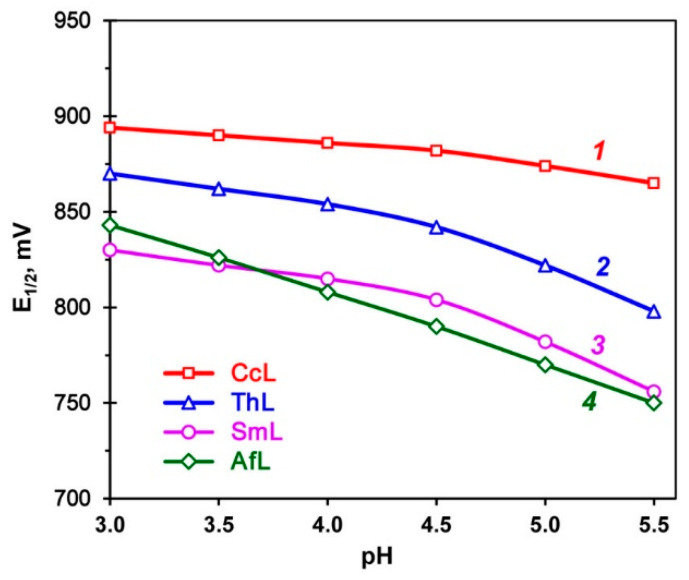
Plots of the change in E_1/2_ as a function of pH values for various laccases: *1*—CcL; *2*—ThL; *3*—SmL; *4*—AfL.

We analyzed the dependence of E_1/2_ on the pH value (Figure 2). For all the laccases studied, we observed a linear decrease in the E_1/2_ value with a slope of 10–35 mV/pH in a pH range of 3.0–4.5. For the ThL and SmL, there was a deviation from the linear dependence at pH > 4.5.

The current model of bioelectrocatalysis [28] states that, in the case of MCOs, the electrode replaces the reductant substrate acting as an electron donor. Consequently, the mechanism of electrocatalytic reaction (the electron transfer from the electrode through the T1 center to the T2/T3 cluster) is expected to resemble homogeneous catalysis. Therefore, in the present work, we examined the catalytic properties of laccases in homogeneous oxidation reactions of substrates with different values of redox potentials. To rule out the influence of non-enzymatic reactions, which usually accompany the enzymatic oxidation of organic substrates (donors of hydrogen atoms), we used electron donor substrates such as K_4_Fe(CN)_6_, K_4_Mo(CN)_8_, and ABTS (Table 3). It should be noted that the K_M_ values for oxygen in laccase-catalyzed reactions were in the range of 150–200 μM [45], which allowed us to carry out kinetic studies in air-saturated buffer solutions.

Upon oxidation of the high-redox-potential substrate K_4_Mo(CN)_8_ (E^0^ = 782 mV [46]), the catalytic efficiency of laccases (k_cat_/K_M_) at pH 5.5 decreased as follows: CcL > AfL > ThL > SmL (Table 3). In the case of ABTS (E^0^ = 682 mV [47]) and K_4_Fe(CN)_6_ (E^0^ = 412 mV [48]) oxidation, the laccases exhibited a different catalytic efficiency: AfL ≥ CcL > ThL > SmL and CcL > SmL ≥ AfL > ThL, respectively. A number of studies have shown that in homogeneous reactions, the efficiency of laccase-catalyzed oxidation of various organic substrates (hydrogen atom donors), expressed in lg(k_cat_/K_M_), correlates with the difference between the potentials of the laccase T1 center and the substrate (ΔE = E^0^_T1_ − E^0^_S_) [49,50,51]. Our results demonstrated the lack of such a correlation in the oxidation of electron donor substrates (Supplementary Figure S2).

Glazunova et al. [52] also showed the absence of this correlation in the laccase-catalyzed oxidation of hydrogen atom donor substrates using CcL, ThL, SmL, and AfL. However, as shown in another study [53], when the high-redox-potential laccases from *T. hirsuta* and *Trametes versicolor* (E^0^_T1_ = 780 and 800 mV) were compared to low-redox-potential laccases from *Rhus vernicifera* and *Melanocarpus albomyces* (E^0^_T1_ = 410 and 460 mV), the correlation of oxidation efficiency of inorganic substrates (electron donors) with the ΔE value was revealed. Yet, no such correlation was observed in the case of phenolic organic substrates (hydrogen atom donors) [53]. Certain high-redox-potential laccases were shown to have differences in the efficiency of the oxidation of phenolic substrates (hydrogen atom donors) and K_4_Fe(CN)_6_ [54]. In the present work, the high-redox-potential CcL had K_4_Mo(CN)_8_ and K_4_Fe(CN)_6_ oxidation efficiency an order of magnitude higher than ThL, with comparable ABTS oxidation efficiency. The medium-redox-potential AfL demonstrated an efficiency of K_4_Mo(CN)_8_ and ABTS oxidation an order of magnitude higher than SmL, with comparable K_4_[Fe(CN)_6_] oxidation efficiency (Table 3).

The absence of a correlation between ΔE = E^0^_T1_ − E^0^_S_ and the efficiency of substrate oxidation in homogeneous reactions might arise from the protein structures of different laccases. Indeed, a number of studies have shown that the efficiency of laccase catalysis in homogeneous reactions depends not only on the T1 center potential, but also on other enzyme characteristics, such as the protein architecture, hydrophobicity, and charge of the substrate-binding pocket [52,55,56,57].

**Figure 3:**
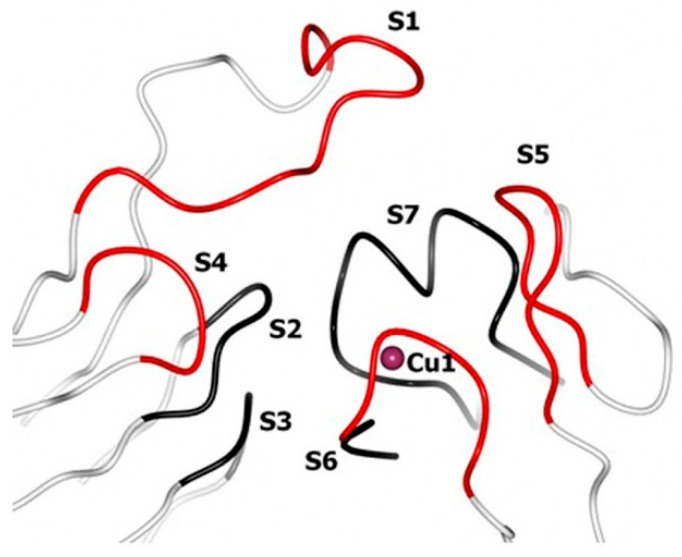
The environment of the T1 center copper ion using the example of the SmL (PDB 5E9N). Structurally conserved loops are shown in black, and variable loops are shown in red.

**Figure 4:**
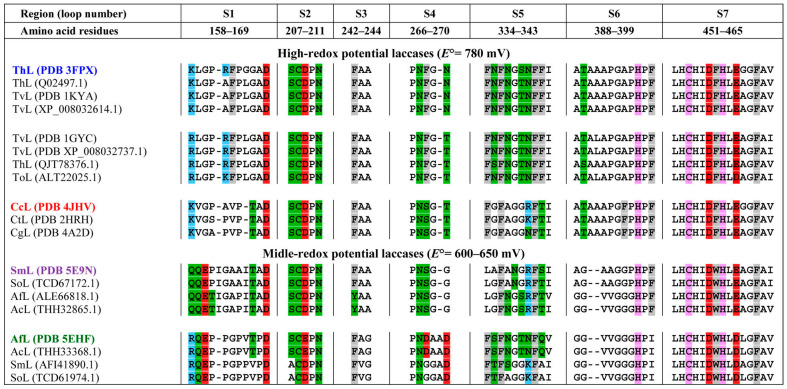
Amino acid sequences of the loops that form the substrate-binding pocket of high- and medium-redox-potential laccases from different orthologous groups (SmL 5E9N laccase numbering). Amino acid residues are highlighted depending on their charge and polarity: positively charged, negatively charged, polar uncharged, and non-polar uncharged aromatic residues are shown in blue, red, green, and gray, respectively. The residues of the first coordination sphere of the T1 center copper ion are highlighted in pink. ThL—*Trametes hirsuta*; TvL—*Trametes versicolor*; ToL—*Trametes ochracea*; CcL—*Coriolopsis caperata*; CtL—*Coriolopsis trogii*; CgL—*Coriolopsis gallica*; SmL—*Steccherinum murashkinskyi*; SoL—*Steccherinum ochraceum*; AfL—*Antrodiella faginea*; AcL—*Antrodiella citronella*.

Glazunova et al. [41] demonstrated that the secondary coordination sphere of the T1 center of fungal laccases consists of seven loops, designated as S1–S7. Loops S2, S3, and S7 are quite conservative, whereas S1, S4, S5, and S6 show a high degree of variability. Figure 3 shows the location of these loops relative to the T1 center copper ion, using the example of SmL (PDB 5E9N). The amino acid composition of these loops in CcL, ThL, SmL, and AfL, as well as laccases from the corresponding orthologous groups, is shown in Figure 4. The amino acid sequence identity of laccases within the same orthologous group is about 80–99%, while the sequence identity between different orthologous groups mostly does not exceed 70%.

Molecular dynamics simulations reported in [55] demonstrated that the amino acid residues Asp209 and His458 (Figure 4) in the conserved S2 and S7 loops of the *T. versicolor* laccase (PDB 1GYC), respectively, play an important role in substrate binding. These amino acid residues are critically important since they form hydrogen bonds, salt bridges, or π-π stacking interactions upon substrate binding. The electron transfer may involve the nitrogen atom of Nε2 His458. Moreover, the ionization potential, shape, and binding affinity of the substrate largely determine the enzyme K_M_ value for a specific substrate [55].

The His458 (H458) residue in loop S7 is highly conserved in all laccases since it is involved in coordinating the T1 center copper ion. The residue at position 209 in the conserved S2 loop is Asp (D209) in the case of ThL and CcL, and SmL and Glu (E209) in the case of AfL (Figure 4). The isoelectric point of Glu is shifted to a more alkaline region (pI 3.22) compared to Asp (pI 2.77). Protonation of the amino acid residue at position 209 affects the mobility of the loops in the substrate-binding pocket [55].

**Figure 5:**
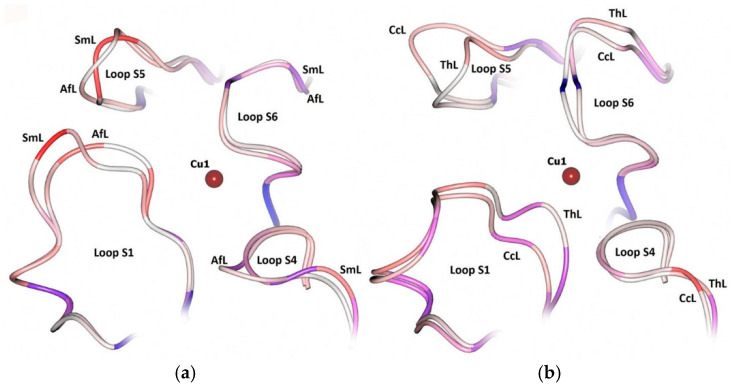
Solvent accessibility of variable loops S1, S4, S5, and S6 near the T1 center of (**a**) medium- (SmL, AfL) and (**b**) high- (CcL, ThL)-redox-potential laccases. Blue—the least accessible regions, red—the most accessible regions.

Variable loops S1, S4, S5, and S6 of the T1 copper ion environment differ in length and amino acid composition in laccases from different orthologous groups (Figure 4). A number of studies [55,56,57] have shown that the structure of the S1 loop, which exhibits high flexibility and significant conformational variability, affects the substrate binding near the T1 center. The S1 loop can adopt three main conformations (hook-like, expanded, and vertical pseudothree-centered arch-like conformations) upon binding to the phenolic substrate catechol [57]. The length and amino acid composition of this loop affect substrate binding and the K_M_ value. Residues Asn267 (N267) and Phe268 (F268) in the S4 loop of the *T. versicolor* laccase (PDB 1GYC) also have a major effect on substrate binding near the T1 center [55]. As can be seen from Figure 4, Asn267 (N267) is highly conserved. At the same time, the laccases studied in this work contained different amino acid residues at position 268: ThL–Phe (F268), CcL and SmL–Ser (S268), and AfL–Asp (D268). Differences in the amino acid composition and length of variable loops S1, S4, S5, and S6 of the four laccases studied apparently affect the charge and hydrophobicity of the loops that form the substrate-binding pocket near the T1 center. As can be seen from Figure 5, the accessibility of the variable loops to the solvent varies, with the most significant differences observed for loops S1 and S5.

The total charge of the loops which form the substrate-binding pocket near the T1 center varies in laccases from different orthologous groups (Figure 4). Loop S1 has a charge of 0, +1, −1, and −2 in CcL, ThL, AfL, and SmL, respectively. The total charge of loop S4 in the AfL is −2; it is 0 in the other three laccases. Loop S5 in CcL and SmL has a positive charge (+1) and it is uncharged in ThL and AfL.

Apart from the potential of the T1 center of laccases, the structure and composition of the loops that form the substrate-binding pocket significantly affect the efficiency of homogeneous catalysis by laccases. This can explain the lack of correlation between the potential of the T1 center of the enzymes studied and their catalytic properties in the homogeneous reactions of electron donor substrate oxidation. In case of heterogeneous catalysis, the effect of the substrate-binding pocket structure on the electrocatalytic characteristics of laccases seems to be negligible, while the T1 center redox potential is a major factor that determines the efficiency of direct bioelectrocatalysis.

## 3. Materials and Methods

### 3.1. Materials

All commercially available chemicals were of high purity and were used without further purification, namely, 2,2′-azino-bis(3-ethylbenzothiazoline-6-sulfonic acid) diammonium salt (ABTS), K_4_Fe(CN)_6_, K_4_Mo(CN)_8_ (Sigma-Aldrich, Steinheim, Germany), citric acid, Na_2_HPO_4_, and NaOH (Riedel-de Haën, Seelze, Germany). The multi-walled carbon nanotubes “Taunit M” (Nano-TechCentre Ltd., Tambov, Russia) (outer diameter 10–30 nm; length ≥ 2 μm; specific surface area ≥ 270 m^2^/g) were used without any modification. Flexible graphite foil (thickness 0.2 mm) was purchased from (Unichimtek, Klimovsk, Moscow region, Russia). All the solutions were prepared using water purified with a Simplicity^®^ Water Purification System (Merck KGaA, Darmstadt, Germany).

### 3.2. Laccases

The mycelial cultures of the basidiomycete strains used in this study were received from the Collection of the Komarov Botanical Institute, Russian Academy of Sciences (St. Petersburg, Russia): *T. hirsuta* strain LE-BIN 072, *C. caperata* strain LE-BIN 0677, *S. murashkinskyi* LE-BIN 1963, and *A. faginea* LE-BIN 1998. Laccases CcL (Lac2; GenBank #AGE13770.1), ThL (LacA; GenBank #AIZ72721.1), SmL (Lac2; GenBank #AFI41889.1), and AfL (LacA; GenBank #ALE66817.1) were isolated from culture liquids of basidial fungi as described in [41,58]. All the enzymes were homogeneous according to SDS electrophoresis. The amino acid sequence of laccase isozymes was confirmed by MALDI-TOF/TOF MS/MS using a BrukerUltraflex II MALDI TOF/TOF mass spectrometer (BrukerDaltonics, Bremen, Germany) [41]. The PDB codes of CcL, ThL, SmL, and AfL structures in Protein Data Bank are 4JHV, 3FPX, 5E9N, and 5EHF, respectively.

### 3.3. Fabrication of Laccase-Modified MWCNT Electrodes

Strips of graphite foil (0.5 × 2 cm) were used as a current collector. A total of 100 μL of MWCNT dispersion in ethanol (1 mg mL^−1^) was applied to the surface of carbon foil without a binder and dried in air. The electrode working surface was 0.25 cm^2^. An enzyme solution was applied to the MWCNT-electrode, and after 30 min the electrodes were washed several times with 0.1 M Na–citrate–phosphate buffer to remove the loosely binding enzyme. The maximum enzyme adsorption on the MWCNT’s surface was achieved under these conditions.

The surface concentration of laccases adsorbed on the MWCNT-electrodes (Γ, pmol cm^−2^) was evaluated based on the difference between the protein concentrations in the solution before and after enzyme adsorption. Protein concentration was determined as described in [59] by the difference in the optical density of the protein solution at 228.5 and 234.5 nm using bovine serum albumin (BSA) as a protein concentration standard.

### 3.4. Kinetic Studies of the Laccases

Kinetic measurements were performed using a Shimadzu UV 1240 mini spectrophotometer (Shimadzu Europa GmbH, Duisburg, Germany). Reactions were carried out at 23 °C in 0.1 M Na–citrate–phosphate buffer at pH 5.5. For each laccase, the range of substrate concentrations and a working enzyme concentration were selected. The following molar extinction coefficients of the ABTS, K_4_Mo(CN)_8_, and K_4_Fe(CN)_6_ oxidation products were used for calculations: ε_420_ = 36,000 M^−1^cm^−1^ [60], ε_388_ = 1400 M^−1^cm^−1^ [61], and ε_420_ = 1040 M^−1^cm^−1^ [62], respectively. The kinetic parameters of the reactions were calculated using the Lineweaver Burk plots. The initial reaction rates were determined at least in triplicate.

### 3.5. Electrochemical Measurements

Electrochemical measurements were performed using a BAS CV-50W voltammetric analyzer (Bioanalytical Systems Inc., West Lafayette, IN, USA) and a single-compartment, three-electrode cell. A platinum wire and an Ag/AgCl electrode (BAS) served as counter and reference electrodes, respectively. MWCNT-covered graphite foil with adsorbed laccase served as a working electrode. Three working electrodes were fabricated for each enzyme. Linear voltammograms were recorded using a stationary electrode in air-saturated 0.1 M Na–citrate–phosphate buffer solution. All potentials are given vs. normal hydrogen electrode (NHE). Results are presented as the mean ± standard deviation. Statistical comparisons are performed using one-way ANOVA followed by Tukey’s post hoc test (*p* < 0.05).

### 3.6. Enzyme Structure Analysis

Laccase amino acid sequence alignment was carried out using ClustalW. The solvent accessible surface area was calculated using service PISA (via CCP4 v9.0.010) (protein interfaces, surfaces, and assemblies) [63].

## 4. Conclusions

We examined the direct bioelectrocatalytic reduction of dioxygen on MWCNT-electrodes modified with high-redox-potential laccases from *Coriolopsis caperata* and *Trametes hirsuta* as well as medium-redox-potential enzymes from *Steccherinum murashkinskyi* and *Antrotiella faginea*. We revealed a correlation between the redox potential of the T1 center and catalytic efficiency of the laccases studied in heterogeneous (electrochemical) reactions. No correlation was observed in the homogeneous reactions of electron donor substrate oxidation. This can be explained by differences in the secondary coordination sphere of the T1 copper ion, namely, the amino acid composition of the conservative and variable loops responsible for substrate binding. Therefore, for practical applications of laccases, their structure and catalytic properties should preliminary be studied in detail.

## Figures and Tables

**Table 1:** Some biochemical properties of laccases.

Laccase	Mw, kDa	pI	Carbohydrate Content, %	E^0^_T1_, mV *	Copper Content	Ref.
CcL	63	3.5	16	780 ± 10	4	[40]
ThL	66	4.0	12	780 ± 10	4	[40]
SmL	63	3.0	9	650 ± 20	4	[41]
AfL	65	4.5	17	620 ± 50	4	[41]

* vs. NHE.

**Table 2:** Some characteristics of MWCNT-electrodes with adsorbed laccases.

Laccase	Γ, pmol cm^−2^	E_ss_, * mV	E_onset_, * mV	E_½_, * mV	Δi/ΔE, μA mV^−1^
CcL	1.86 ± 0.31	856 ± 16 ^a^	843 ± 11 ^a^	818 ± 9 ^a^	1.65
ThL	1.67 ± 0.23	830 ± 18 ^ab^	818 ± 14 ^ab^	789 ± 11 ^a^	1.43
SmL	1.58 ± 0.25	809 ± 19 ^ab^	787 ± 13 ^bc^	742 ± 10 ^b^	0.54
AfL	1.81 ± 0.20	792 ± 18 ^b^	776 ± 10 ^c^	747 ± 7 ^b^	0.66

* Measurements were performed in 0.1 M Na–citrate–phosphate buffer, pH 5.5, at 23 °C. All the potentials are given vs. NHE. Means within the same column with different superscripts are significantly different (*p* < 0.05).

**Table 3:** Kinetic parameters of laccase-catalyzed oxidation of electron donor substrates *.

Laccase	K_4_Mo(CN)_8_ E_0_ = 782 mV [46]			ABTS E_0_ = 682 mV [47]			K_4_Fe(CN)_6_ E_0_ = 412 mV [48]		
	k_cat_, s^−1^	K_M_, μM	k_cat_/K_M_, s^−1^ μM^−1^	k_cat_, s^−1^	K_M_, μM	k_cat_/K_M_, s^−1^ μM^−1^	k_cat_, s^−1^	K_M_, μM	k_cat_/K_M_, s^−1^ μM^−1^
CcL	74.6 ± 3.3	334.7 ± 19.4	0.223	73.2 ± 3.9	11.8 ± 0.5	6.20	81.8 ± 3.3	50.4 ± 2.8	1.62
ThL	23.7 ± 2.4	1685.0 ± 56.9	0.014	74.7 ± 3.1	16.7 ± 0.2	4.47	65.6 ± 3.2	245.1 ± 7.2	0.27
SmL	9.3 ± 0.3	1270.3 ± 48.6	0.007	33.3 ± 1.4	71.7 ± 2.9	0.46	62.7 ± 1.9	90.0 ± 2.7	0.70
AfL	20.6 ± 0.4	480.0 ± 22.1	0.043	32.7 ± 1.1	4.9 ± 0.4	6.67	57.2 ± 1.6	88.1 ± 6.4	0.65

* Measurements were performed in 0.1 M Na–citrate–phosphate buffer, pH 5.5, at 23 °C. The substrate potentials are given vs. NHE.

## Data Availability

The original contributions presented in this study are included in the article. Further inquiries can be directed to the corresponding authors.

## Supplementary Materials

The following supporting information can be downloaded at: https://www.mdpi.com/article/10.3390/ijms26157488/s1.

## Abbreviations

The following abbreviations are used in this manuscript:

MCO	Multicopper oxidase
TNC	Trinuclear copper cluster
MWCNT	Multi-walled carbon nanotube
NHE	Normal hydrogen electrode
ABTS	Diammonium salt of 2,2′-azino-bis(3-ethylbenzothiazoline-6-sulfonic acid)
ThL	*Trametes hirsuta* laccase
CcL	*Coriolopsis caperata* laccase
SmL	*Steccherinum murashkinskyi* laccase
AfL	*Antrodiella faginea* laccase
TvL	*Trametes versicolor* laccase
ToL	*Trametes ochracea* laccase
CtL	*Coriolopsis trogii* laccase
CgL	*Coriolopsis gallica* laccase
SoL	*Steccherinum ochraceum* laccase
AcL	*Antrodiella citronella* laccase
