# Supplementary material for: Fungal Laccases with High and Medium Redox Potential: Is the T1 Center Potential a Key Characteristic of Catalytic Efficiency in Heterogeneous and Homogeneous Reactions?
Source: Int J Mol Sci. 2025 Aug 2;26(15):7488. doi: 10.3390/ijms26157488 (PMC12347638; doi:10.3390/ijms26157488)
Supplement: Supplementary file 1 [file ijms-26-07488-s001.zip › ijms-3775526-supplementary.pdf]

Supplementary Materials

Fungal laccases with high and medium redox potential: is the T1 center potential a key characteristic of catalytic efficiency in heterogeneous and homogeneous reactions?

Olga Morozova *, Maria Khlupova, Irina Vasil'eva, Alexander Yaropolov and Tatyana Fedorova *

[1] A. N. Bach Institute of Biochemistry, Research Center of Biotechnology of the Russian Academy of Sciences, Leninsky Ave. 33, 119071 Moscow, Russia; dave80@yandex.ru (M.K.); ir-vas@yandex.ru (I.V.); yaropolov@inbi.ras.ru (A.Y.)
* Correspondence: fedorova_tv@mail.ru (T.F.); morozova@inbi.ras.ru (O.M.)

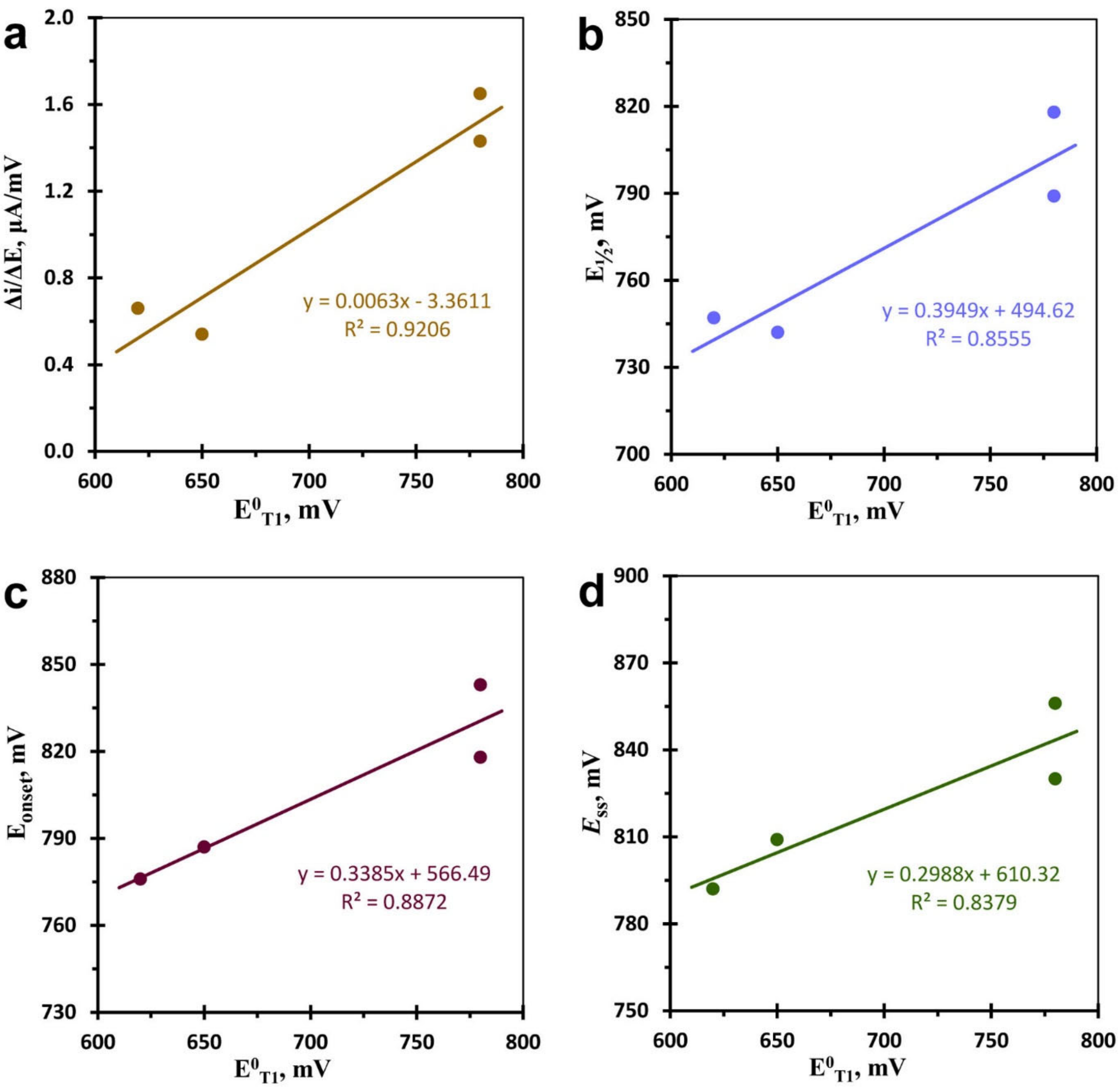

Figure S1. Correlation of $\Delta i/\Delta E$ (**a**), $E_{1/2}$ (**b**), E_{onset} (**c**), E_{ss} (**d**) with the T1 center redox potential (E^0_{T1}) of laccases.

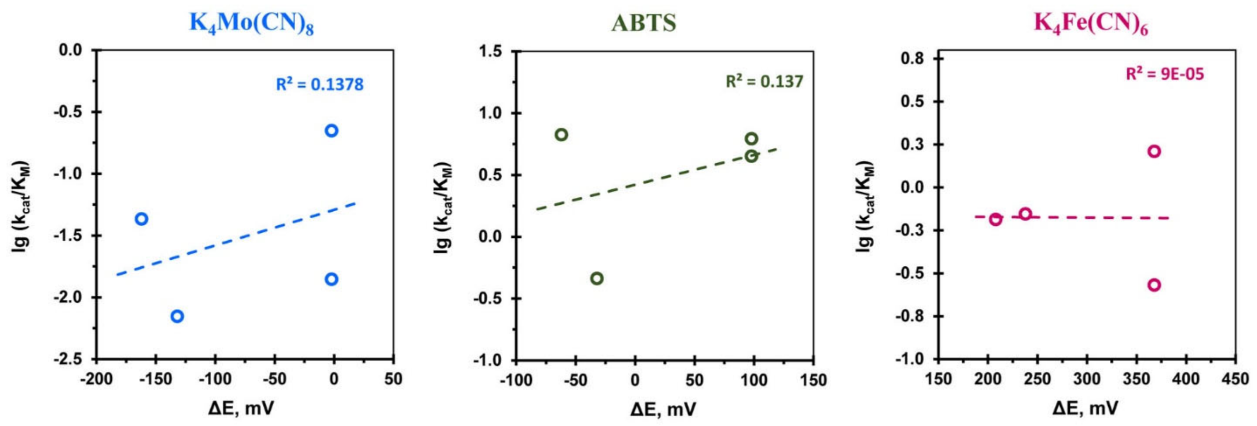

Figure S2. Plots of lg(k_{cat}/K_M) as a function of difference between potentials of the laccase T1 center and substrate ($\Delta E = E^0_{T1} - E^0_S$).
